# Supplementary material for: A Comparative Analysis of Usual- and Gastric-Type Cervical Adenocarcinoma in a Japanese Population Reveals Distinct Clinicopathological and Molecular Features with Prognostic and Therapeutic Insights
Source: Int J Mol Sci. 2025 Aug 1;26(15):7469. doi: 10.3390/ijms26157469 (PMC12347983; doi:10.3390/ijms26157469)
Supplement: Supplementary file 1 [file ijms-26-07469-s001.zip › ijms-3775165-supplementary.pdf]

A Comparative Analysis of Usual- and Gastric-Type Cervical Adenocarcinoma in a Japanese Population Reveals Distinct Clinicopathological and Molecular Features with Prognostic and Therapeutic Insights

Umme Farzana Zahan, Hasibul Islam Sohel, Kentaro Nakayama, Masako Ishikawa, Mamiko Nagase, Sultana Razia, Kosuke Kanno, Hitomi Yamashita, Shahataj Begum Sonia and Satoru Kyo

Supplementary Table S1. Univariate and multivariate analyses of progression-free survival using a Cox proportional hazards model in patients with p16 in usual-type cervical adenocarcinoma.

Factors	Patients (n = 94)	Univariate			Multivariate		
		HR	95% CI	*p*-Value	HR	95% CI	*p*-Value
**Age**							
< 50 years	62	0.532	0.236–1.293	0.156			
≥ 50 years	32	ref					
**Clinical FIGO stage**							
I, II	69	0.194	0.081–0.464	0.001	0.76	0.252–2.87	0.053
III, IV	25	ref			ref		
**Lymph node involvement**							
Yes	15	ref	1.04-3.12	0.045	ref	0.165–1.86	0.438
No	79	1.79			0.553		
**Distant metastasis**							
Yes	2	ref	1.25-10.1	0.018	ref	0.009–0.589	0.014
No	92	3.56			0.066		
**Vaginal invasion**							
Yes	13	ref			ref		
No	81	6.765	0.853–51.71	0.063	5.311	0.469–50.77	0.149
**p16**							
Negative	12	ref			ref		
Positive	82	0.376	0.122–0.83	0.023	0.765	0.231–2.82	0.714

Supplementary Table S2. Univariate and multivariate analyses of overall survival using a Cox proportional hazards model in patients with p16 in usual-type cervical adenocarcinoma.

Factors	Patients (n = 94)	Univariate			Multivariate		
		HR	95% CI	*p*-Value	HR	95% CI	*p*-Value
**Age**							
< 50 years	62	0.58	0.233–1.446	0.243			
≥ 50 years	32	ref					
**Clinical FIGO stage**							
I, II	69	0.202	0.087–0.565	0.002	0.912	0.253–3.289	0.659
III, IV	25	ref			ref		
**Lymph node involvement**							
Yes	15	ref	0.87-3.21	0.12			
No	79	1.64					
**Distant metastasis**							
Yes	2	ref	0.004–0.223	0.095	ref	0.88-6.84	0.066
No	92	0.026			1.71		
**Vaginal invasion**							
Yes	13	ref			ref		
No	81	4.756	0.75–8.32	0.202	1.8	0.508–7.86	0.55
**p16**							
Negative	12	ref			ref		
Positive	82	0.409	0.164–1.020	0.036	0.965	0.231–2.82	0.548

Supplementary Table S3. Univariate and multivariate analyses of progression-free survival using a Cox proportional hazards model in patients with PD-L1 in gastric-type cervical adenocarcinoma.

Factors	Patients (n = 16)	Univariate			Multivariate		
		HR	95% CI	*p*-Value	HR	95% CI	*p*-Value
**Age**							
< 50 years	9	0.59	0.203–1.46	0.264			
≥ 50 years	7	ref					
**Clinical FIGO stage**							
I, II	10	0.222	0.087–0.565	0.002	ref	0.246–3.89	0.095
III, IV	6	ref			0.886		
**Lymph node involvement**							
Yes	4	ref	0.201–1.35	0.043	ref	0.157–2.32	0.542
No	12	0.655			0.532		
**Distant metastasis**							
Yes	1	ref	0.002–0.293	0.006	ref	0.008–1.25	0.056
No	15	0.024			0.112		
**Vaginal invasion**							
Yes	6	ref			ref		
No	10	6.765	0.903–50.705	0.059	5.311	0.549–51.377	0.149
**PD-L1**							
Positive	3	3.22	1.63–7.54	0.008	2.28	1.10–6.75	0.014
Negative	13	ref			ref		

Supplementary Table S4. Univariate and multivariate analyses of overall survival using a Cox proportional hazards model in patients with PD-L1 in gastric-type cervical adenocarcinoma.

Factors	Patients (n =16)	Univariate			Multivariate		
		HR	95% CI	*p*-Value	HR	95% CI	*p*-Value
**Age**							
< 50 years	9	0.452	0.236–1.30	0.151			
≥ 50 years	7	ref					
**Clinical FIGO stage**							
I, II	10	0.163	0.013–0.475	0.035	ref		
III, IV	6	ref			1.011	0.320–3.87	0.86
**Lymph node involvement**							
Yes	4	ref	0.162–.089	0.029			
No	12	0.279					
**Distant metastasis**							
Yes	1	ref	0.008–0.764	0.018	ref	0.032–4.95	0.527
No	15	0.087			0.544		
**Vaginal invasion**							
Yes	6	ref			ref		
No	10	4.756	0.725–9.32	0.202	1.8	0.508–7.86	0.55
**PD-L1**							
Positive	3	2.83	1.00–5.01	0.029	1.99	1.14–3.92	0.018
Negative	13	ref			ref		

Supplementary Table S5. Description of primary antibodies for IHC.

Antibody name	Sources	Dilution ratio
p53 (DO-7)	Dako	1:50
p16	VENTANA (Roche)	1:2
PD-L1	AdipoGen Life Science	1:150
PD-1	AdipoGen Life Science	1:150
CD8 (SP57)	VENTANA (Roche)	1:3
ARID1A (PSG3)	Santa Cruz Biotechnology INC.	1:100
ARID1B (2D2)	Abcam	1:100
c-Myc (9E10)	Santa Cruz Biotechnology INC.	1:100
PTEN (138G6)	Cell Signaling Technology	1:200

Supplementary Table S6. Primer sequences used in mutational analysis.

Gene	Forward	Reverse
KRAS exon 2	TTAACCTTATGTGTGACATGTTCTAA	AGAATGGTCCTGCACCAGTAA
PIK3CA exon 9	GGGAAAAATATGACAAAGAAAGC	CTGAGATCAGCCAAATTCAGTT
BRAF exon 15	TGCTTGCTCTGATAGGAAAATG	AGCATCTCAGGGCCAAAAAT
